# Microbiological profile of slow-growing non-tuberculous mycobacteria species other than *Mycobacterium avium* complex

**DOI:** 10.3389/fmicb.2025.1572162

**Published:** 2025-04-25

**Authors:** Mariana Fernandez-Pittol, Sara Batista, Sofía Narváez, Angely Román, Lorena San Nicolás, Diego Martínez, Laura Oliver, Olga González-Moreno, José Antonio Martínez, Felipe García, Rosanel Amaro-Rodríguez, Néstor Soler, Amadeu Gené, Araceli González-Cuevas, Griselda Tudó, Julian Gonzalez-Martin

**Affiliations:** ^1^Servei de Microbiologia, CDB, Hospital Clínic de Barcelona, Barcelona, Spain; ^2^Departament de Fonaments Clínics, Facultat de Medicina i Ciències de la Salut, Universitat de Barcelona, Barcelona, Spain; ^3^ISGLOBAL, Institute for Global Health, Barcelona, Spain; ^4^Fundació de Recerca Clínic Barcelona –Institut d’Investigacions Biomèdiques August Pi i Sunyer (FRCB-IDIBAPS), Barcelona, Spain; ^5^Catlab - Centre Analítiques Terrassa AIE, Servei de Microbiología/Vallés Occidental Parc Logístic de Salut Vial Sant Jordi, Barcelona, Spain; ^6^Departamento de Microbiología y Parasitología, SYNLAB Diagnósticos Globales, Barcelona, Spain; ^7^Servei de Malalties Infeccioses, Hospital Clínic-Universitat de Barcelona, Barcelona, Spain; ^8^CIBER of Infectious Diseases (CIBERINFEC), Instituto de Salud Carlos III, Madrid, Spain; ^9^Department of Pneumonology, Hospital Clínic-Universitat de Barcelona, Barcelona, Spain; ^10^Laboratori, Hospital Sant Joan De Deu, Barcelona, Spain

**Keywords:** non-tuberculous mycobacteria, identification, drug susceptibility test, *in vitro* profile, treatment, MIC value

## Abstract

**Introduction:**

*Mycobaterium avium* complex (MAC) and *Mycobacterium abscessus* complex are the primary agents of non-tuberculous mycobacteria infection. However, other species within the slow-growing group can also be potentially pathogenic, although information on these species is limited.

**Objectives:**

We conducted a prospective analysis of slow-growing species other than MAC, aimed at the identification and microbiological profiles of clinical samples from a tertiary hospital. The Microbiology Department of the Hospital Clinic of Barcelona, the Microbiology Laboratory of SYNLAB Laboratories, and the Microbiology Laboratory of Hospital Sant Joan de Deu participated in the study.

**Methods:**

Species identification was conducted by MALDI-TOF MS and/or *16S rRNA* and *rpo*B gene sequencing. Drug susceptibility tests (DST) were performed using the microdilution method. The results of the susceptibility profiles were compared with treatment guidelines, or the most recent literature related to each species.

**Results:**

Twenty-five different species belonging to the slow-growing group were identified. The most frequently observed were *M. xenopi, M. kansasii*, *M. gordonae*, and *M. marinum*. In this series, *M. lentiflavum* presented the highest susceptibility profile, while *M. simiae* demonstrated the highest level of resistance. Clarithromycin, rifabutin, and amikacin demonstrated high levels of effectiveness across all species. The species most associated with infection, presented a high correlation with the clinical treatment guidelines.

**Conclusion:**

A specific susceptibility profile was observed among all the species. The *in vitro* profiles of the most frequent species correlated with the clinical treatment guidelines, reinforcing the supporting role of DST in the design of individualized treatment for each patient.

## Introduction

Infections by non-tuberculous mycobacteria (NTM) have increased in the last years (Iseman and Marras, 2008). To date, more than 170 species have been described in the *Mycobacterium* genus (Baldwin et al., 2019). These species of NTM are usually classified into two groups: slow-growing NTM (with a growth rate ≥ 7 days on solid subculture) and rapid-growing NTM (with a growth rate < 7 days on solid subculture) with *Mycobacterium abscessus* complex (MAB) being a representative species of this group (Baldwin et al., 2019). Within the category of slow-growing NTM, three sub-groups can be identified. These include photochromogen species, such as *Mycobacterium kansasii* or *Mycobacterium marinum*; scotochromogen species, such as *Mycobacterium scrofulaceum,* and non-chromogen species, mainly *Mycobacterium avium* complex (MAC; Baldwin et al., 2019). However, in this latter sub-group, other less representative species, which are also related to human infection, such as *Mycobacterium xenopii, Mycobacterium simiae* or *Mycobacterium malmoense*, can be found (Baldwin et al., 2019).

In general, infections caused by NTM are primarily produced by the species of MAC and MAB. Nevertheless, other species of the slow-growing NTM group can also be considered as potentially pathogenic. Accurate identification, monitoring and, if possible, drug susceptibility tests (DST) are essential for managing NTM infectious, although treatment guidelines often lack cut-off points (Baldwin et al., 2019; Haworth et al., 2017). Additionally, in cases in which an NTM species is isolated, it is important to establish its clinical relevance and rule out host colonization or lab contamination before starting treatment (Baldwin et al., 2019). Therefore, multidisciplinary management is required to ensure adequate patient care (Haworth et al., 2017).

Risk factors for NTM diseases have been described and differ depending on the site of infection. Individuals with a pre-existent lung condition, particularly those with bronchiectasis, are at the highest risk (Haworth et al., 2017). However, NTM species can also have a predilection for other organs depending on the specific species. For instance, *Mycobacterium ulcerans* or *M. marinum* are associated with skin infection, while *Mycobacterium chelonae* or *Mycobacterium fortuitum* are associated with soft tissue infection and disseminated diseases are commonly caused by MAC. Despite this knowledge, accurately identifying a case caused by species other than MAC is challenging (Haworth et al., 2017). The decision to initiate treatment, especially when these species are involved, is complex (Haworth et al., 2017; Yan et al., 2023). Prolonged therapy is often required, which may lead to secondary effects that may not always be accompanied by adequate response by the host (Yan et al., 2023). Furthermore, conducting DST also poses a challenge. Guidelines offer recommendations for specific species and antibiotics but the cut-off points for microorganisms other than MAC are limited (Yan et al., 2023). Additionally, data related to these species in terms of epidemiology, diagnosis, susceptibility patterns and management are scarce, presenting a persistent hurdle (Haworth et al., 2017; Yan et al., 2023). The present study aimed to describe the microbiological profiles of different species of the slow-growing NTM group other than MAC in clinical samples.

## Materials and methods

Microbiological analysis was performed prospectively over an 8.5-year period (January 2013 to June 2021). Three microbiology laboratories participated in the collection and culture of the samples: the Microbiology Department of the Hospital Clinic of Barcelona (MDHC), the Microbiology Laboratory of SYNLAB Laboratories, and the Microbiology Laboratory of Hospital Sant Joan de Deu. Final identification and DST were centralized in the MDHC. The clinical samples analyzed were collected from patients during diagnostic procedures or follow-up controls. Mycobacterial culture was simultaneously performed on solid Löwenstein–Jensen medium (Becton Dickinson, Franklin Lakes, NJ, United States) and in liquid BD BACTEC mycobacteria growth indicator tubes (BACTEC MGIT 960 system, Becton Dickinson) according to the manufacturer’s instructions.

### Microbiological identification

Isolate identification was performed by matrix-assisted laser desorption ionization time of flight mass spectrometry (MALDI-TOF MS Bruker, Bremen, Germany) following a previously described protocol (Rodriguez-Temporal et al., 2022). The isolates identified as belonging to other species, such as *M. kansasii, Mycobacterium gordonae, M. marinum, M. scrofulaceum* and *M. xenopi*, were also confirmed by amplification and sequencing of *16S rRNA* and *rpo*B genes from liquid culture (Devulder et al., 2005; De Zwaan et al., 2014).

### Drug susceptibility testing

DST was performed using a commercial microdilution method Sensititre™ Myco SLOMYCOI AST plate (Thermo Fisher Scientific, Massachusetts, United States) following the manufacturer’s instructions. The analysis of the study was focused on 12 antibiotics: amikacin, ciprofloxacin, clarithromycin, cotrimoxazole, doxycycline, ethambutol, ethionamide, linezolid, moxifloxacin, rifabutin, rifampicin, and streptomycin. The break points of resistance were established according to the Clinical & Laboratory Standards Institute guidelines (Woods et al., 2011; Brown-Elliott and Woods, 2019). The break points of antibiotics not included in these guidelines were based on those described in the literature (Soni et al., 2016). For *M. kansasii* and *M. xenopii*, isoniazid was also analyzed. The established break point for considering a strain as susceptible was <1 μg/mL (Alcaide et al., 2004). DST quality control was performed using the *M. avium* ATCC 25291 reference strain, which was tested monthly throughout the study period.

### Statistical analysis

Frequency data was described by sex, age, sample type, isolate identification, and minimum inhibitory concentration (MIC) values. Species with fewer than five isolates were excluded from the susceptibility profile analysis because insufficient statistical power to reliably demonstrate the species profile pattern. Categorical data were expressed by number and percentages for each drug among the species. All the calculations were made using Rstudio package version 4.0.5. Additionally, the *in vitro* susceptibility profiles of each species were compared with the antibiotic activity and their treatment recommendations as reported in the literature or treatment guidelines. Three categories were used for comparison: high correlation, when three or more of the recommended drugs showed high activity (between 80% and 100%) in *in vitro* results; moderate correlation, when one or two of the recommended drugs showed high activity (between 80 and 100%) in *in vitro* results; and low correlation, when none of the recommended drugs showed high activity (between 80 and 100%) in *in vitro* results.

**Figure 1:**
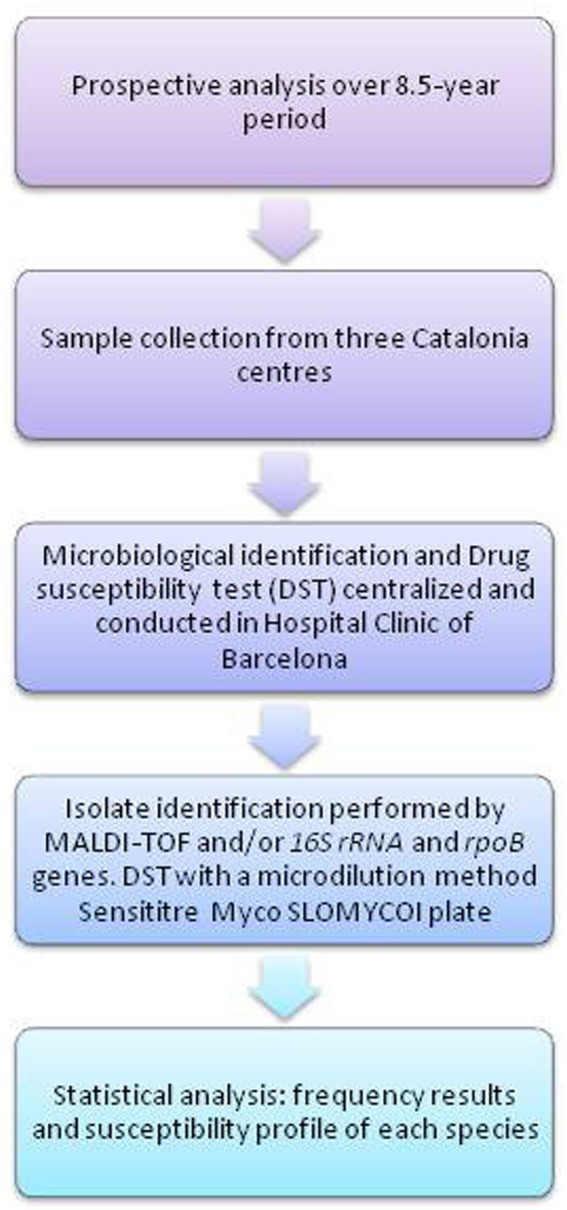
Flow chart of study design.

Figure 1 show a flow chart illustrating the study design, outlining the different steps followed to obtain the results.

## Results

During the study period, a total of 1,141 isolates were identified as slow-growing NTM. Of these, 857 (75.1%) were classified as MAC and 284 (24.8%) were identified as species other than MAC. Off these isolates, 86 (30.2%) samples were collected and analyzed in the MDHC, while the remaining 198 (69.7%) samples were collected from the other two centers. The 284 isolates were obtained from 220 patients. A total of 159 (55.9%) were pulmonary samples, among which 110 were sputum samples, 47 were bronchial aspirate samples, and the remaining two were from tracheal aspiration and bronchoalveolar lavage. The remaining 125 (44.0%) comprised extrapulmonary specimens, sourced from various origins, including: lymph nodes, cutaneous biopsies, pleural fluid, ascites fluid, abscess, gastric fluid, synovial fluid, faces, and bone marrow. Eighty-five (38.6%) samples were obtained from females and 135 (61.3%) from males. The median age of this cohort was 58.5 years (interquartile range 72.25–34.25).

### Microbiological results

Sixty-four strains (22.5%) were identified as *M. xenopi,* 46 (16.1%) as *M. kansasii*, 20 (7.0%) *M. marinum*, 14 (4.9%) *M. scrofulaceum,* 14 (4.9%) *Mycobacterium arupense*, 12 (4.2%) *Mycobacterium lentiflavum,* 11 (3.8%) *Mycobacterium parascrofulaceum*, 10 (3.5%) *M. malmoense*, 9 (3.1%) *M. simiae*, 9 (3.1%) *Mycobacterium kumamotonense*, 8 (2.8%) *Mycobacterium celatum,* 7 (2.4%) *Mycobacterium terrae,* 4 (1.4%) *Mycobacterium interjectum*, and 3 (0.9%) *Mycobacterium florentinum*. Two strains (0.7%) of each species, including *Mycobacterium bohemicum, Mycobacterium colombiense, Mycobacterium paragordonae* and *Mycobacterium triplex* were identified. Additionally, one (0.3%) of each species of *Mycobacterium branderi, Mycobacterium cospiccum, Mycobacterium gastri, Mycobacterium genavense, Mycobacterium hassiacum,* and *Mycobacterium yongonense* was also identified. Finally, 39 (13.7%) were identified as *Mycobacterium gordonae.*

**Figure 2:**
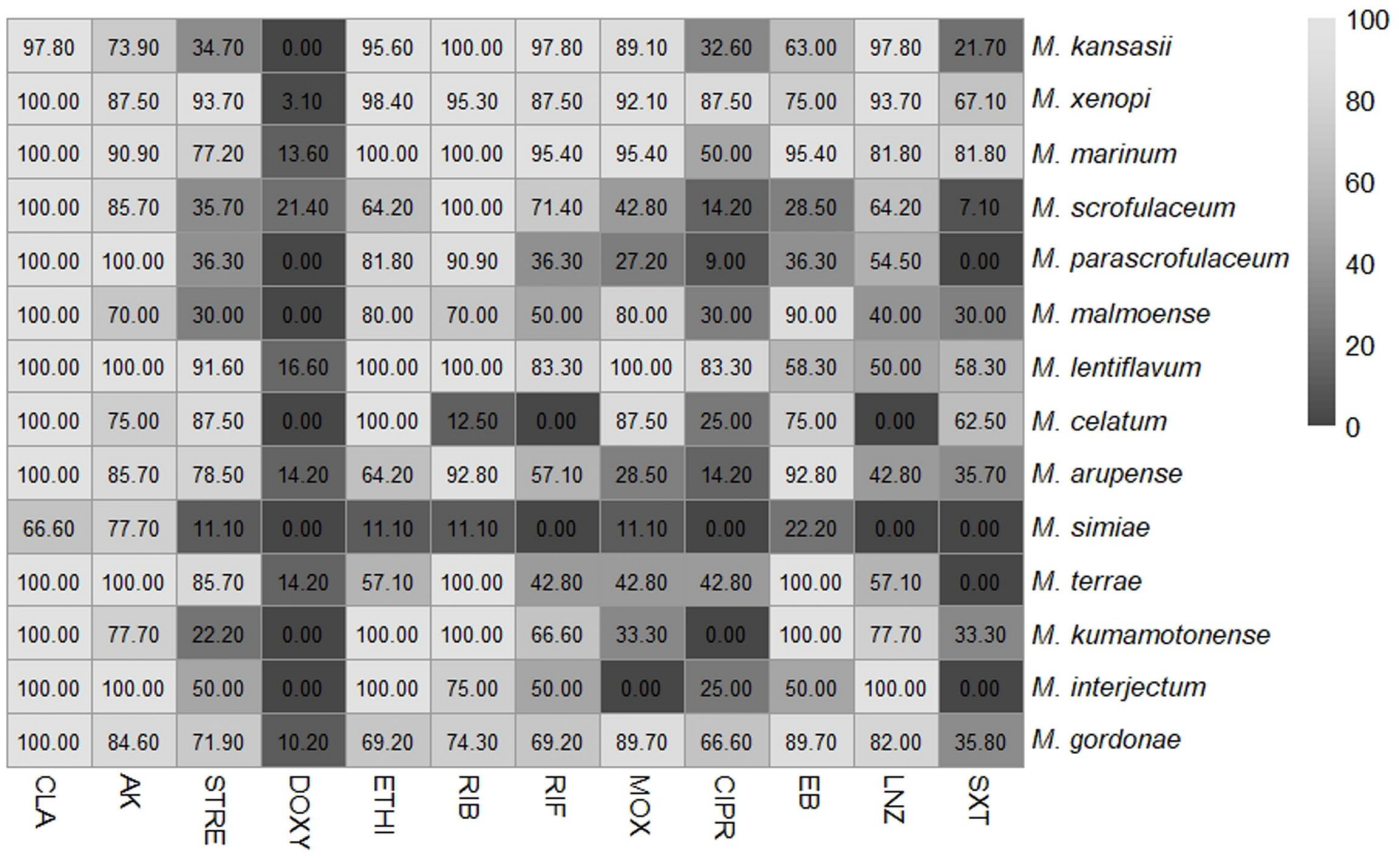
Percentage of susceptible isolates for each antibiotic assessed among several slow-growing non-tuberculous mycobacteria (NTM) different from *Mycobacterium avium* complex. The numbers represent the percentage of susceptible isolates. NTM-species (total isolates): *M. kansasii* (46), *M. xenopi* (64), *M. marinum* (20), *M. scrofulaceum* (14), *M. parascrofulaceum* (11), *M. malmoense* (10), *M. lentiflavum* (12), *M. celatum* (8), *M. arupense* (14), *M. simiae* (9), *M. terrae* (7), *M. kumamotonense* (9), *M. interjectum* (4) and *M. gordonae* (39). CLA, clarithromycin; AK, amikacin; STREP, streptomycin; DOXY, doxycycline; ETHI, ethionamide; RIB, rifabutin; RIF, rifampicin; MOX, moxifloxacin; CIPRO, ciprofloxacin; EB, ethambutol; LNZ, linezolid; SXT, cotrimoxazole.

Figure 2 shows the percentage of drug susceptibility of all species with four or more isolates. *M. lentiflavum* showed the most susceptible profile and *M. simiae* the most resistant. The results based on susceptibility or resistance to the 12 antibiotics evaluated are presented as follows: clarithromycin, showed susceptibility to most of the species, apart from a strain of *M. kansasii* and two of *M. simiae*. Quinolones displayed a different pattern, with ciprofloxacin showing a higher resistance compared to moxifloxacin. For aminoglycosides half of the species demonstrated resistance to streptomycin, while over 80% of the strains across all the evaluated species were susceptible to amikacin. Rifamycins showed high activity, especially rifabutin, which presented a more favorable profile across all the species except for *M. celatum* and *M. simiae*, with over 80% of the strains exhibiting resistance to this antibiotic. Linezolid demonstrated a predominantly favorable susceptibility pattern for almost all the strains, except *M. malmoense*, *M. celatum*, *M. arupense* and *M. simiae*, where 60% or more of the strains demonstrate resistance to this antibiotic. Variations were also observed when considering the distribution of the MIC values across all the species analyzed. Table 1 summarizes the MIC values of the six species with greatest number of isolates identified (*M. kansasii*, *M. xenopi*, *M. marinum*, *M. scrofulaceum*, *M. arupense*, and *M. gordonae*).

**Table 1:** Minimum inhibitory concentration (MIC) distribution results of 12 antibiotics against the most frequently isolated of the slow growing species in the present study.

		*M. kansasii*	*M. xenopi*	*M. marinum*	*M. scrofulaceum*	*M. arupense*	*M. gordonae*
ATB	MIC (μg/mL)	Total strain (%)	Total strain (%)	Total strain (%)	Total strain (%)	Total strain (%)	Total strain (%)
CLA	≤2	43 (93.4)	60 (93.7)	19 (95.0)	14 (100.0)	14 (100.0)	35 (89.7)
	4–8	2 (4.3)	2 (3.1)	1 (5.0)	0 (0.0)	0 (0.0)	4 (10.2)
	16	0 (0.0)	2 (3.1)	0 (0.0)	0 (0.0)	0 (0.0)	0 (0.0)
	≥32	1 (2.1)	0 (0.0)	0 (0.0)	0 (0.0)	0 (0.0)	0 (0.0)
AK	≤2	7 (15.2)	49 (76.5)	14 (70.0)	3 (21.4)	5 (35.7)	28 (71.7)
	4–16	39 (84.7)	14 (21.8)	6 (30.0)	11 (78.5)	7 (50.0)	11 (28.2)
	32	0 (0.0)	0 (0.0)	0 (0.0)	0 (0.0)	0 (0.0)	0 (0.0)
	≥64	0 (0.0)	1 (1.5)	0 (0.0)	0 (0.0)	2 (14.2)	0 (0.0)
STRE	≤4	17 (36.9)	61 (95.3)	5 (25.0)	4 (28.5)	10 (71.4)	28 (71.7)
	8	13 (28.2)	0 (0.0)	14 (70.0)	7 (50.0)	1 (7.1)	4 (10.2)
	>16	16 (34.7)	3 (4.6)	1 (5.0)	3 (21.4)	3 (21.4)	7 (17.9)
DOXY	≤2	0 (0.0)	1 (1.5)	1 (5.0)	3 (21.4)	1 (7.1)	5 (12.8)
	4–8	2 (4.3)	7 (10.9)	1 (5.0)	0 (0.0)	1 (7.1)	12 (30.7)
	≥16	44 (95.6)	56 (87.5)	18 (90.0)	11 (78.5)	12 (85.7)	22 (56.4)
ETHI	≤2.5	45 (97.8)	61 (95.3)	20 (100.0)	9 (64.2)	8 (57.1)	22 (56.4)
	4–10	0 (0.0)	2 (3.1)	0 (0.0)	0 (0.0)	4 (28.5)	8 (20.5)
	>10	1 (2.1)	1 (1.5)	0 (0.0)	5 (35.7)	2 (14.2)	9 (23.0)
RIF	≤1	45 (97.8)	56 (87.5)	19 (95.0)	10 (71.4)	8 (57.1)	28 (71.7)
	2–8	1 (2.1)	4 (6.2)	1 (5.0)	4 (28.5)	6 (42.8)	7 (17.9)
	>8	0 (0.0)	4 (6.2)	0 (0.0)	0 (0.0)	0 (0.0)	4 (10.2)
RIB	≤1	46 (100.0)	61 (95.3)	20 (100.0)	14 (100.0)	14 (100.0)	29 (74.3)
	2–8	0 (0.0)	3 (4.6)	0 (0.0)	0 (0.0)	0 (0.0)	9 (23.0)
	>8	0 (0.0)	0 (0.0)	0 (0.0)	0 (0.0)	0 (0.0)	1 (2.5)
EB	≤2.5	2 (4.3)	10 (15.6)	19 (95.0)	3 (21.4)	14 (100.0)	28 (71.7)
	4–8	33 (71.7)	40 (62.5)	1 (5.0)	3 (21.4)	0 (0.0)	7 (17.9)
	≥16	11 (23.9)	14 (21.8)	0 (0.0)	8 (57.1)	0 (0.0)	4 (10.2)
MOX	≤2	41 (89.1)	59 (92.1)	19 (95.0)	6 (42.8)	4 (28.5)	37 (94.8)
	4–8	3 (6.5)	3 (4.6)	1 (5.0)	8 (57.1)	5 (35.7)	2 (5.1)
	>8	2 (4.3)	2 (3.1)	0 (0.0)	0 (0.0)	5 (35.7)	0 (0.0)
CIPR	≤2	31 (67.3)	60 (93.7)	19 (95.0)	3 (21.4)	3 (21.4)	31 (79.4)
	4–8	9 (19.5)	2 (3.1)	1 (5.0)	2 (14.2)	6 (42.8)	5 (12.8)
	≥16	6 (13.0)	2 (3.1)	0 (0.0)	9 (64.2)	5 (35.7)	3 (7.6)
LNZ	≤8	45 (97.8)	60 (93.7)	19 (95.0)	11 (78.5)	6 (42.8)	34 (87.1)
	16	1 (2.1)	1 (1.5)	1 (5.0)	3 (21.4)	0 (0.0)	3 (7.6)
	≥32	0 (0.0)	3 (4.6)	0 (0.0)	0 (0.0)	8 (57.1)	2 (5.1)
SXT	≤2	10 (21.7)	43 (67.1)	18 (90.0)	1 (14.2)	5 (35.7)	14 (35.8)
	4–8	10 (21.7)	6 (9.3)	1 (5.0)	4 (28.5)	0 (0.0)	9 (23.0)
	>8	26 (56.5)	15 (23.4)	1 (5.0)	9 (64.2)	9 (64.2)	16 (41.0)

*Species: (number of isolates): M. kansasii* (46), *M. xenopi* (64), *M. marinum* (20), *M. scrofulaceum* (14), *M. arupense* (14), *and M. gordonae* (39); ATB, antibiotic; CLA, clarithromycin; AK, amikacin; STREP, streptomycin; DOXY, doxycycline; ETHI, ethionamide; RIF, rifampicin; RIB, rifabutin; EB, ethambutol; MOX, moxifloxacin; CIPRO, ciprofloxacin; LNZ, linezolid; SXT, cotrimoxazole.

In the analysis of isoniazid susceptibility, 86.9% of *M. kansasii* and 89.0% of *M. xenopii* strains were found to be susceptible to this drug. The distribution of the MICs was as follows: *M. kansasii* 0.25–1 μg/mL and for *M. xenopii* < 0.25–1 μg/mL.

Finally, Table 2 shows the profile results of each species compared to current treatment recommendations (Daley et al., 2020; Haworth et al., 2017). Five species were excluded from the table due to limited information in the literature. These species were *M. arupense* (high-efficacy drugs: clarithromycin, amikacin, rifabutin and, ethambutol); *M. kumamotonense* (high-efficacy drugs: clarithromycin, rifabutin, ethionamide and ethambutol); *M. parascrofulaceum* (high-efficacy drugs: clarithromycin, amikacin, ethionamide, and rifabutin); *M. celatum* (high-efficacy drugs: clarithromycin, streptomycin, ethionamide and moxifloxacin) and *M. interjectum* (high-efficacy drugs: clarithromycin, amikacin, ethionamide and linezolid). The only data available for these species were from case reports.

**Table 2:** Profiles of slow-growing non-tuberculous mycobacteria other than MAC and the current treatment recommendations.

NTM-species	*In-vitro* susceptibility profile: high activity drugs (80%–100%)	*In-vitro* susceptibility profile: medium activity drugs (60%–79%)	Treatment recommendations	Author (year of publication)	Correlation between *in-vitro* profile vs. guideline recommendations^*^
*M. kansasii*	CLA, ETHI, RIB, RIF, MOX, INH and LNZ.	AK, EB	Rifampicin susceptible: regimen of rifampicin, ethambutol and isoniazid or macrolide. Rifampicin resistant: ethambutol, azithromycin, and fluoroquinolone.	Official ATS/ERS/ESCMID/IDSA Treatment recommendation (2019) (Daley et al., 2020).	High correlation
			Rifampicin sensitive: regimen of rifampicin, ethambutol and isoniazid or macrolide. Rifampicin-resistant: three-drug regimen guided, but not dictated by drug susceptibility test results using a daily oral regimen.	British Thoracic Society guidelines for the management of NTM pulmonary disease (2017) (Haworth et al., 2017).	High correlation
*M. xenopi*	CLA, AK, ETHI, RIB, RIF, MOX, CIP, INH and LNZ.	EB, SXT	Regimen of at least 3 drugs: rifampicin, ethambutol and a macrolide or a fluroquinolone. In severe cases, it is suggested to add parental amikacin.	Official ATS/ERS/ESCMID/IDSA Treatment recommendation (2019) (Daley et al., 2020).	High correlation
			A four-drug regimen (where tolerated): rifampicin, ethambutol, and a macrolide (clarithromycin or azithromycin), with either a quinolone (ciprofloxacin or moxifloxacin) or isoniazid. In severe cases an injectable aminoglycoside (amikacin or streptomycin) should be considered.	British Thoracic Society guidelines for the management of NTM pulmonary disease (2017) (Haworth et al., 2017).	High correlation
*M. marinum*	CLA, AK, ETHI, RIB, RIF, MOX, EB and SXT.	STREP	The choice of the therapy seemed to be based on personal experience. Clinical reports showed different antibiotic regimens including monotherapy with cyclines or combination of sulfamethoxazole and trimethoprim, rifampicin, and ethambutol. Rarely, clarithromycin, levofloxacin, and amikacin. In severe cases surgical debridement is recommended	Aubry A, et al. Conducted a study involving 63 cases (2023) (Mazzarelli et al., 2024).	High correlation
*M. scrofulaceum*	CLA, AK and RIB.	ETHI, LNZ	Clarithromycin or azithromycin combined with one or two other in vitro active drugs (e.g., a fluoroquinolone, linezolid, amikacin, rifamycin with or without ethambutol)	Wilson J, et al. Conducted a study involving 17 cases (2019) (Aubry et al., 2002).	High correlation
*M. malmoense*	CLA, ETHI, MOX, and EB.	AK, RIB	Azithromycin (or clarithromycin), rifampicin, and ethambutol. Fluoroquinolones (moxifloxacin or levofloxacin), clofazimine, or aminoglycosides can be used in case of intolerance or drug resistance to macrolides, rifamycin’s, or ethambutol.	Consensus management recommendation for less common non-tuberculous mycobacterial pulmonary disease (2022) (Lange et al., 2022).	Moderate correlation
*M. lentiflavum*	CLA, AK, STREP, ETHI, RIB, RIF, MOX, CIP		Experience from case reports considered clarithromycin, rifabutin and ethambutol.	Miqueleiz-Zapatero A, et al. Conducted a study involving 23 pediatric cases (2018) (Smith et al., 2000).	Moderate correlation
*M. simiae*		AK, CLA.	Azithromycin (clarithromycin), Moxifloxacin, Clofazimine, Trimethoprim plus Sulfamethoxazole, amikacin IV for severe or cavitary disease.	Consensus management recommendation for less common non-tuberculous mycobacterial pulmonary disease (2022) (Lange et al., 2022).	Low correlation
*M. terrae*	CLA, AK, STREP, RIB and EB.		Experience from case reports considered regimens of combination of rifampicin, ethambutol, and macrolide.	Smith S, et al. Conducted a study involving 54 cases (2000) (Wilson et al., 2019).	High correlation
*M. gordonae*	CLA, AK, MOX, EB and LNZ.	STREP, ETHI, RIB, RIF, CIPRO	Treatment just in case of overwhelming evidence of disease. Regimen that included clarithromycin, rifampicin, and ethambutol.	Consensus management recommendation for less common non-tuberculous mycobacterial pulmonary disease (2022) (Lange et al., 2022).	Moderate correlation

NTM, non-tuberculous mycobacterial; IV, intravenous; CLA, clarithromycin; AK, amikacin; STREP, streptomycin; DOXY, doxycycline; ETHI, ethionamide; RIB, rifabutin; RIF, rifampicin; MOX, moxifloxacin; CIPRO, ciprofloxacin; EB, ethambutol; LNZ, linezolid; SXT, cotrimoxazole. ^*^Each category is described in Materials and methods.

## Discussion

In our series, a total of 25 different species belonging to the slow-growing NTM group were identified, using mainly MALDI-TOF MS to differentiate among species. Among these species, *M. xenopi, M. kansasii*, *M. gordonae*, and *M. marinum* presented the highest number of identifications with 20 or more isolates each.

In regard to DST, there is currently no universal standardized protocol. Specific recommendations have been proposed for some species, but the topic remains controversial. In the slow-growing NTM group, correlations have been established for macrolides and amikacin in MAC lung disease and for rifampicin and clarithromycin in *M. kansasii* lung disease (Daley et al., 2020). Our results showed that all the species identified presented high susceptibility to clarithromycin. These results align with the recommended use of a macrolide as an effective antibiotic in this group (Daley et al., 2020; Lange et al., 2022). Even in the study by Mazzarelli et al. (2024), where the phenotypic and molecular antibiogram were compared, the effectiveness of the macrolide in the different species of NTM could be established.

The different species analyzed in the present study, showed specific species drug profiles. *M. lentiflavum* exhibited the most susceptible profile, while *M. simiae* demonstrated the highest level of resistance. Although both species belong to the *M. simiae* complex, information regarding *M. lentiflavum* is scarce, whereas *M. simiae* is well recognized for its inherent *in vitro* natural drug resistance (Lange et al., 2022). This study suggests that these differences may be due to the morphologic characteristics specific to each specie.

Based on the criteria of susceptibility or resistance to the different drugs analyzed against the species of the slow-growing NTM group identified, our findings indicate that clarithromycin, rifabutin and, amikacin demonstrate high levels of effectiveness across all species. Moxifloxacin and linezolid also showed moderate to high effectiveness against most species. In contrast, doxycycline showed the lowest activity, maintaining some activity againts *M. scrofulaceum*, *M. lentiflavum*, *M. arupense*, *M. terrae* and *M. marinum*, with a 13% to 20% of strains remaining susceptible.

In relation to rifamycin’s and quinolones, *in vitro* results showed that rifamycins exhibited low MICs among the species, although some variability among species was observed. Rifabutin demonstrated greater activity when compared to rifampicin with lower resistance rates. As for quinolones, moxifloxacin was consistently more favorable than ciprofloxacin in terms of MICs. The variation among species highlighted the need to understand the antibiotic activity profiles, underscoring the importance of accurate species identification and DST analysis in these species.

In regard to the literature and guideline recommendations, a high correlation was observed between *in vitro* results and treatment recommendations for the most important species causing infection, such as *M. kansasii*, *M. xenopi*, *M. marinum*, and *M. scrofulaceum* (Daley et al., 2020; Haworth et al., 2017; Aubry et al., 2002; Wilson et al., 2019). A high correlation has been observed for other species, such as *M. terrae,* which are commonly associated with colonization and/or contamination (Smith et al., 2000).

In the case of *M. simiae*, which was identified as the most resistant species, a low correlation was found with their *in vitro* profile results, for which the antibiotic recommendations by the consensus guidelines presented medium effectiveness (60%–79%) (Lange et al., 2022). The rest of the species, including *M. malmoense, M. lentiflavum,* and *M. gordonae*, presented a moderate correlation, compared with the consensus guidelines (Lange et al., 2022). Although relying on series reports is a limitation for making conclusions, the scarce data on these species makes it challenging to use other comparisons to assess clinical response (Miqueleiz-Zapatero et al., 2018).

It was of note that the species most frequently associated with infection, generally presented a high correlation with the treatment recommendation based on clinical outcomes. This reinforces the clinical recommendations and also enhances the reliability of the *in vitro* results and highlight the value of the DST findings and their supporting role in treatment decision making.

Finally, our study involved the identification and analysis of drug profiles of various species aside from MAC. The different species analyzed showed specific drug profiles on DST with certain similarities among them. Three antibiotics, clarithromycin, amikacin and rifabutin, were found to be highly active against all the species. Two other antibiotics, moxifloxacin and linezolid, were also moderately to highly active against these species.

The drug susceptibility profile of the most frequent species isolated in the present study showed a high correlation with the treatment schedules recommended in clinical guidelines and the literature, demonstrating the supporting role of DST in the design of individualized treatment for each patient.

## Data Availability

The raw data supporting the conclusions of this article will be made available by the authors, without undue reservation.
